# Chemical-nutritional characteristics and aromatic profile of milk and related dairy products obtained from goats fed with extruded linseed

**DOI:** 10.5713/ajas.18.0868

**Published:** 2019-04-15

**Authors:** Francesca Bennato, Andrea Ianni, Denise Innosa, Lisa Grotta, Andrea D’Onofrio, Giuseppe Martino

**Affiliations:** 1 Faculty of Bioscience and Technology for Food, Agriculture and Environment, University of Teramo, 64100 Teramo, Italy

**Keywords:** Extruded Linseed, Ripened Goat Cheeses, Fatty Acids, Volatile Compounds

## Abstract

**Objective:**

This study aimed to evaluate the effect of dietary integration with extruded linseed (EL) on fatty acid (FA) and aromatic profile of goat cheese after 60 (T_60_) days of ripening.

**Methods:**

Thirty goats were divided in two groups. The control group (CG) was fed with conventional diet, whereas the experimental group (EL+) was fed with conventional diet supplemented with 10% of EL. Milk samples were collected on 30 and 60 days of trial to determinate chemical-nutritional composition and FA profile. At the end of experiment, six cheese-making sessions (3 for each group) were carried out using a pooled milk sample obtained from the 15 goats of each group. At 60 days of ripening, cheeses were analyzed for chemical-nutritional composition, FA and aromatic profile.

**Results:**

An increase in the milk production, protein, fat and lactose were evidenced in the EL+ goats. Conversely, a reduction of somatic cells was observed in the EL+ compared with the CG. However, no variation was observed for urea and casein levels content in milk samples, and no changes in protein and lipid content were found for cheeses in the two experimental groups. Dietary supplementation with EL modified the FA profile of milk. There was a decrease in saturated FAs and an increase in polyunsaturated FAs. Chemical composition of T_60_ cheese did not differ between the two groups but a different FA profile was observed. In T_60_ cheese obtained from EL+ milk, an increase in short-chain FA and a decrease in medium and long-chain FA were observed. The EL diet led to cheeses with butanoic acid 2 times higher compared to CG cheeses. Moreover, a greater presence of aldehyde compounds and alcohols were observed in the cheeses of experimental group.

**Conclusion:**

The present study pointed out that EL supplementation may improve the chemical and physical qualities of goat milk and cheeses.

## INTRODUCTION

In recent years, a renewed interest has been observed for the lipidic integration in ruminant rations which in addition to animal diet can increase the productive performances, improve the health conditions and the nutritional characteristics of animal products. It has been demonstrated that the nature of fat content of sheep and goat milk, in comparison with cow milk, presents several benefits for consumer health. This composition makes them foods of interest for segments of the human population with specific requirements. Different nutritional strategies have been designed with the aim of optimizing the composition of these milks with respect to their fat content and composition. Experimental results in goat nutrition, showed that lipid supplementation does not change net energy intake and milk yield but increases, in most cases, milk fat content and allows much less saturated fatty acids (SFA), much more oleic and/or vaccenic and rumenic acids, more linolenic acid, and other trans fatty acids (FAs). The responses of goats are different from cow’s responses in many aspects of milk production and mammary lipid metabolism, and the same is probably true for sheep [1]. In this regard, several studies have been conducted on nutritious foods, such as linseeds [2,3]. Linseed have nutritional characteristics and are rich source of ω-3 fatty acid: α-linolenic acid, polyunsaturated fatty acids (PUFA), soluble and insoluble fibers, proteins and antioxidants. Extruded linseed (EL) compared to other seeds used for zootechnical feeding, are the most effective in increasing the concentration of α-linolenic acid and its isomers. Experiments conducted on ruminant have highlighted that a supplementation with linseed cause an increase in milk PUFA and the same concentrations have been found in the cheese [4,5]. Feeding with linseed is also reflected in a lower ratio of ω6/ω3, suggesting that such lipid supplementation would improve cheese FA profiles [6]. Milk fat is important in the genesis of cheese properties and a great number of cheese aroma compounds originate from lipid degradation. To date, few studies have addressed the effect of EL supplementation on FA and volatile compounds (VOCs) profile of cheese produced using goat milk. Thus, the present study aimed to investigate the possible effect of dietary integration with EL on FA and aromatic profile of goat cheese after 60 days of ripening.

## MATERIALS AND METHODS

### Animals, experimental design and diet

The experimental plan was performed according to Directive 2010/63/EU of the European Parliament (European Union, 2010) and Directive 86/609/EEC (European Economic Community, 1986), which deal with the protection of animals used for scientific purposes. In this study no animal sacrifices were conducted.

Thirty Saanen goats, homogeneous for age, weight, lactation, number of parts, productivity and body condition, have been used in this study. Animals have been divided into two groups of fifteen goats each: a control group (CG) and an experimental group (EL+) whose diet was supplemented with EL. The study was conducted for 60 days, the goats were housed for the entire trial period in two separate areas of free housing with bunks on straw, a drinking trough and an access to an identical feeding area. In this period, each groups received *ab libitum* first-cutting alfalfa hay, whose chemical composition is reported in Table 1 and 1.2 kg/head/d of total mixed ration (TMR).

Samples of TMR, as reported in Table 2, were analyzed for dry matter (DM; method 930.15), crude protein (CP; method 954.01), ether extract (method 920.39), crude fiber (method 962.09), and ash (method 942.05) according to AOAC methods [7]; neutral detergent fiber, acid detergent fiber, and acid detergent lignin were determined by the detergent procedures of Van Soest et al [8].

The CG received, in the form of “unifeed” and during the entire test, a complete food formulated taking into account the nutritional needs of cows in mid-lactation. The EL+ received the same complete food, formulated according to the same requirements and prepared in the same way, however integrating the ration with 10% of EL.

### Milk yield, composition and fatty acids profile

On 30 and 60 days of trial, individual and bulk milk samples were collected. Samples of 50 mL each, collected in triplicate were partly immediately analyzed for composition and partly stored at −20°C for FA assessment. Chemical composition of milk (fat, protein, casein, lactose, urea) was determined by MilkoScan FT 6000 (Foss, Hillerød, Denmark), and somatic cells content (SCC) was determined using a Fossomatic TM FC (Foss, Denmark). Milk lipid fraction was extracted according to the AOAC official method [7] and the extracted fat was analyzed for FA composition. Separation of fatty acid methyl esters (FAME) was performed by GC using a gas chromatograph (Thermo Scientific, Waltham, MA, USA) equipped with a capillary column (Restek Rt-2560 Column fused silica 100 m×0.25 mm highly polar phase; Restek Corporation, Bellefonte, PA, USA) and a flame ionization detector (FID). Hydrogen was used as carrier gas. The initial holding temperature was 55°C for 1 min; then it was increased to 170°C at a rate of 10°C/min and held for 30 min. The final temperature of 215°C was reached at a rate of 2°C/min and was held for 4 min. Peak areas were quantified using ChromeCard software, and the relative value of each individual FA was expressed as a percentage of the total FA.

### Cheese making

After 60 days of dietary integration, the bulk milk of 4 consecutive milking obtained from the two respective groups was conveyed into two separate tanks and subsequently subjected to rennet coagulation. Bulk milk was pasteurized at 72°C for 20 s, cooled to 36°C±1°C, and transferred to a container in which there was the addiction of 500 U/5,000 L of a combination of thermophilic and mesophilic starter cultures (WhiteDaily, Chr Hansen, Hoersholm, Denmark). After acidification there was the addiction of rennet (15 g/100 kg; 75% of chymosin and 25% of pepsin; 1:18,000 strength; Clerici, Cadorago, Italy) to the milk; coagulation began after 30 min of incubation. The curd was broken into small pieces resembling the size of hazelnuts, portioned in aliquots of ca. 1 kg, transferred into plastic moulds and kept at 48°C±1.5°C until the pH reached a value of 5.20±0.1. After this, a 20% NaCl water solution was used to salt the cheese in brine. Thereafter, the salted, fresh cheese was stored in the ripening room at a controlled temperature (10°C±0.5°C) and a relative humidity of 85%. From the cheese-making process, 6 forms of cheese were produced, of about 1 kg each, 3 for the experimental group, and subjected to a maturation process of 60 days.

### Cheese composition and fatty acids analysis

Samples of 20 g each, collected in triplicate from three different cheese-makings, were partly immediately analyzed and partly packed under vacuum and frozen at −20°C until analysis. In the case of cheese, the determination of DM, total proteins and ash were determined according to AOAC International [9]. In order to obtain informations about the proteolytic event in cheese during ripening, fractions of 12% trichloroacetic acid-soluble nitrogen were determined according to Kuchroo and Fox [10] and Polychroniadou et al [11].

The extraction of cheese lipids was carried out by acid hydrolysis with 20 mL of ethanol and 500 μL of hydrochloric acid 3 N. FAME were extracted using a mix of chloroform and methanol (2:1, vol/vol). Seventy mg of lipids were reconstituted with 1 mL of hexane and 500 μL of sodium methoxide in methanol (1:1, vol/vol) was used for methylation. Separation of FAME was performed by GC-FID according to the same condition used for milk FAME analyses.

### Evaluation of lipid oxidation in cheese by thiobarbituric acid reactive substances-test

Lipid oxidation in cheeses was evaluated by measuring thiobarbituric acid reactive substances. The analysis was performed according to the procedure reported by Grotta et al [12]. For each sample, an aliquot of 2.5 g of frozen cheese was mixed, within 2 min of sample withdrawal from the freezer, with 500 μL of 0.1% of butylated hydroxytoluene in methanol to stop the oxidation process. The mixture was homogenized with Ultra Turrax T-25 high speed homogenizer (IKA, Staufen, Germany) in 20 mL of an acqueous solution of 7.5% trichloroacetic acid, and then subjected to distillation. An aliquot of 5 mL of each distillate was mixed with an equal volume of a 0.02 M thiobarbituric acid solution in 90% acetic acid. The solution was kept for 45 min in a thermostated bath at 95°C, and only after cooling, the absorbance at 534 nm was evaluated with a spectrophotometer (Jenway, Essex, UK). The amount of malondialdehyde (MDA) of each sample was calculated by using a calibration curve and results were expressed in μg of MDA per g of cheese.

### Volatile compounds analysis by solid-phase microextration and gas chromatography-mass spectrometry

Extraction of VOCs from cheese samples was performed by solid-phase microextraction (SPME), and gas chromatography-mass spectrometry (GC-MS) analysis was performed with a gas chromatograph (Perkin Elmer, Waltham, MA, USA) coupled with a mass spectrometer (SQ8S; Perkin Elmer, USA). The gas chromatograph was equipped with an Elite-5MS column (length×internal diameter: 30×0.25 92 mm; film thickness: 0.25 μm; Perkin Elmer, USA). Five g of cheese previously grated were mixed with 10 mL of saturated sodium chloride solution (360 g/L), and then 10 μL of internal standard solution (4-methyl-2-heptanone; 10 mg/kg in ethanol) was added. The vials were sealed with a polytetrafluoroethylene-silicone septum (Supelco, Bellefonte, PA, USA) and stirred at 60°C; VOCs were extracted from the headspace with a divinylbenzene-carboxen-polydimethylsiloxane SPME fiber (length: 1 cm; film thickness: 50/30 μm; Supelco, USA) with an exposition time of 60 min. After adsorption time, the extracted VOC were thermally desorbed into the gas chromatograph injector splitless mode for 1 min at 250°C. The oven temperature was held at 50°C for 1 min, increased at a rate of 3°C/min up to 200°C and held for 1 min, and then increased from 200°C to 250°C at 100.15°C/min and held for 15 min. Helium was used as a carrier gas at a flow rate of 1 mL/min. The mass spectrometer operated in electronic impact ionization mode at 70 eV, and data were collected in full scan mode, with a scan time of 0.2 s over a mass range of 35 to 350. Source and interface temperature were held at 250°C. The VOCs were identified by comparison with mass spectra of a library database (NIST Mass Spectral library, National Institute of Standards and Technology, Gaithersburg, MD, USA) and by comparing the eluting order with Kovats indices.

### Statistical analysis

Statistical data processing was performed by the general linear model procedure of the statistical package SPSS 13.0 (SPSS 2006), using a one-way analysis of variance considering the effect of the feeding strategy as factor of variation. When the variance analysis was significant (p<0.05), the differences between the means were compared by the least significant difference test.

## RESULTS

### Chemical composition of milk and cheeses

The dietary integration with EL resulted in a moderate increase in milk production (p<0.05), fat (p<0.01), protein (p<0.05), and lactose (p<0.05) contents (Table 3). The SCC was lower (p<0.05) in EL+ group compared with the CG group. No significative differences in milk urea and casein content were observed between the two groups. Conversely, the chemical-nutritional composition of cheese after 60 (T_60_) days of ripening was not affected by the dietary inclusion of EL (Table 4), the percentage of proteins, lipids, total and soluble N and DM did not change among the two groups.

### Fatty acids profile of milk and cheeses

Table 5 shows how the dietary integration with EL has influenced the FA profile of the milk. In particular, the diet resulted in an increase of butyric acid (C4:0, p<0.05), caproic acid (C6:0, p<0.05) and caprylic acid (C8:0, p<0.05) and a reduction in SFA 10 to 17 carbon atoms. A significant increase (p<0.05) of stearic acid (C18:0), vaccenic acid (C18:1 t11), oleic acid (C18:1 c9), least significant difference (CLA) (C18:2 c9,t11) and α-linolenic acid (C18:3 c9,12,15) have been observed in the EL+. Conversely, linoleic acid (C18:2 c9,12) was significantly higher in the CG than EL+ milk (p<0.05).

Instead, the FA profile of the cheese has been influenced by the diet and it differs from FA milk profile (Table 5). More specifically, an increase in short- and medium-chain FA (SCFA and MCFA) and a decrease in long-chain FA were observed. In particular, the diet resulted in a significant increase of butyric acid (C4:0, p<0.01), caproic acid (C6:0, p<0.01), and caprylic acid (C8:0, p<0.05) and a decrease of palmitic acid (C16:0, p<0.05). The amount of PUFA did not differ between the two groups. No significant changes in lipid oxidation levels were reported. In particular, the concentration of MDA was 2.42±0.07 in CG and 2.02±0.06 μg MDA/g in EL+ respectively.

### Effect of diet on the cheese volatile compounds

Several families of VOCs were detected in T_60_ cheese samples, obtained respectively from animals fed with standard diet and animals subjected to food supplementation with EL (Table 6). The most abundant class of compounds was represented by carboxylic acids. A significant increase of butanoic acid (6.42±0.57; p<0.01) and a decrease of dodecanoic acid (6.30±0.57; p<0.01) were found in the EL+. A general significant increase in aldehydes and alcohols were observed in the EL+ samples compared to the CG. In particular, in EL+ cheeses an increase of nonanale (1.76±0.15; p<0.01), 2–3-butenediol (3.30±0.28; p<0.05), 1-butanol, 3-methyl (22.62±1.96, p<0.01), and phenylethyl alcohol (14.36±1.31) was observed. Conversely a decrease of ketones was found in EL+ compared to CG.

## DISCUSSION

The dietary supplementation with EL influenced milk production (p<0.05) and this effect could be due to the maximum energy content of the integrated ration with linseed compared to the control diet. This increase in milk production could explain the highest percentage of fat (p<0.01), proteins and lactose (p<0.05) found in the CG milk compared to the EL+. The results of the present study are in agreement with most previous results on sheep [13,14]. Chilliard and collaborators have showed that the dietary integration with PUFA in goats does not cause decreases in the lipid content of milk contrary to what happens in dairy cows and this could be due to a lower inhibition by long-chain polyunsaturated fatty acids (LCPUFA) of the enzyme acetyl-CoA carboxylase [15–17]. Relatively proteins, it has been observed that the addition of fats in goat dietary is not sufficient in determining a decrease but it may instead determine an increase, as evidenced by the present work and by Sampelayo and collaborators [14]. In the present study it has been observed in the EL+ a considerable reduction (p<0.01) compared with CG in SCC, our data are in line with that reported by Gantner and Kompan [18]. There are no studies that show how the integration with linseed acts on the SCC, however, some research have demonstrated that the alimentation indirectly influences the SCC. For example, a poor quality food can predispose to infectious and metabolic diseases by increasing the susceptibility of the mammary gland to inflammation. Morgante and collaborators affirm that a dietary supplementation with vitamin A or beta carotene, vitamin E and selenium promotes the immune defense of the mammary gland by reducing the incidence of infections that can induce an increase in somatic milk cells [19,20]. Consequently, we can suppose that linseed contain substances with anti-inflammatory and immunomodulatory activity able to positively affect the state of health of the breast by reducing the SCC of milk.

In this study no variations were observed in urea and casein levels, although some studies reported an increase in milk casein percentage in ewes feed with flaxseed supplementation [21]. Gonthier et al [22] reported that extrusion was ineffective in increasing the post-ruminal supply of amino acids from flaxseed-based diets as a result of an increase in ruminal CP digestibility and a reduction in the amount of CP available for digestion post-ruminally for cows fed diets of extruded flaxseeds. However, the effect of extrusion on ruminal nutrient degradability may vary according to parameters used for heating (e.g., temperature of extrusion and resident time), which may modify the response to feeding extruded flaxseed.

The diet with EL resulted in an increase in milk of butyric acid (C4:0, p<0.05), caproic acid (C6:0, p<0.05), and caprylic acid (C8:0, p<0.05) and a reduction in SFA 10 to 16 carbon atoms. This result can be related to the PUFA inhibiting action on the synthesis of FA in the mammary gland. The FAs C6:0 to C14:0 are in fact synthesized ex novo by the mammary gland, while C16:0 derives partly from the diet and partly from the synthesis ex novo. The linseed supplementation produces a greater quantity of PUFA which compete in the esterification process with SCFA and MCFA. The accumulation of these FAs causes an inhibition of the lipogenic enzymes with consequent reduction in the ex novo synthesis of the FA derived from mammary gland [23]. In line with the data reported in the bibliography, the integration with linseed resulted in a significant increase (p<0.05) of stearic acid (C18:0), vaccenic acid (C18:1 t11), and oleic acid (C18:1 c9). The increase in the levels of stearic and vaccenic acid in the EL+ derives from the PUFA biohydrogenation. In fact, stearic acid is the final product of these biochemical processes while the vaccenic acid is an intermediate product. The stearic acid is converted into the mammary gland by Δ-9 desaturase enzyme in oleic acid (C18:1 c9) this explain the significant increase of this FA in the milk of the treated goats. Despite the low transfer efficiency of ω-3 PUFA from diet to milk fats, the levels of α-linolenic acid in the milk of treated goats increase significantly (p<0.05). The transfer of these substances is strictly dependent on their percentage present in the diet and also on the physical presentation and quality of the flax used. Gomez-Cortes and collaborators showed that an 12% integration with EL cause an 2% of α-linolenic acid increase in the milk of the sheep treated [24]. Conversely, the additions with whole flaxseed less than 2% on DM have not produce a significant increase of α-linolenic acid in treated sheep milk [25]. This demonstrate how the form of oil seeds in the ration is another factor responsible for the increase of α-linolenic acid in milk fat. As it was logical to expect, linoleic acid (C18:2 c9,12) is significantly higher in the milk of the CG (p<0.05) due to the higher share of soy in the control ration. The rumenic acid (CLA c9, t11) increase in the EL+ compared with the CG, this is closely related to the levels of vaccinal acid produced in the rumen: in fact, most of the milk FAs are produced from the endogenous synthesis via Δ-9 desaturase in the mammary gland from the vaccinal acid. High concentrations of CLA in milk can be obtained when high levels of vaccinal acid are generated in the rumen [26].

Chemical composition of T_60_ cheese was not affected by dietary supplementation with EL. These results are in agreement with those obtained in dairy cows [2]. The FA profile of cheese is not identical to that of milk and this could be result of lipolysis due to the activity of the endogenous lipoprotein lipase contained in milk or to the possible inoculation of other enzymes with lipolytic activity. Collins et al. reported that, during ripening, SCFA should be generated as result of lipolysis, thus their relative concentrations would be increased [27]. The development of VOC in cheese are very complex and this is related to milk properties and composition, processing operations and microbial activity. Several families of VOCs were detected in T_60_ cheese samples and the most abundant class of compounds was represented by carboxylic acids, reflecting the marked lipolysis which takes place during cheese ripening. The increase of butanoic acid observed in EL+ T_60_ cheese could be associated with an increase in the lipolysis of triglycerides by microbial enzymes and endogenous milk enzymes [28]. Butanoic acid is considered one of the main determinants of the aroma of aged cheeses, giving rise to strong and pungent odors. On the other hand, dodecanoic acid has no marked effects on the aroma of dairy products, but is an important substrate for milk lipases, whose activity generates shorter chain carboxylic acids. The fact that the concentration of this compound is considerably greater in the control indicates a positive effect of EL on activity of endogenous milk lipases [27]. As for the other families of compounds, it is possible to highlight a general increase in aldehydes and alcohols in the experimental samples compared to the control. The aldehydes are certainly more interesting from the point of view of the aromatic profile, since their presence is associated with a slightly fruity aroma with notes that refer to lemon [29]. The formation of these compounds is associated with enzymatic events on the carbon chains of free FAs present in cheeses. For what concerns methyl ketones, their biosynthesis is mainly attributed to mold metabolism, such as *Penicillium roqueforti*, *Penicillium camemberti*, and *G. candidum* and due to their typical odors and low perception thresholds, are most likely responsible for the characteristic flavor of surface-mold-ripened, and blue-veined cheeses. However, it is also postulated that ketones can be produced by heating milk, or directly from esterification of β-keto acid [27]. Probably certain compounds present in flax seeds had an inhibitory effect on the mechanisms described above.

## CONCLUSION

This work highlighted how the integration with EL influenced various parameters of dairy goat products. Firstly, was evidenced a moderate increase in the milk production of goats fed with EL supplementation, furthermore a decrease of somatic cells were observed, probably thanks to bioactive compounds present in linseed able to improve the animal welfare. The most interesting aspect of our work was found in the analysis of the FA profile for milk. In fact, the EL supplementation lead to a reduction of SFA and an increase in PUFA, reflecting the fact that consumption of these product could have positive effects on human health. Besides of this, the aromatic profile of ripened goat cheese positively affected by dietary linseed intake. In conclusion, this work showed how the integration with linseed definitely improved the chemical and physical qualities of milk and cheese obtained from the treated group but it would however be necessary to verify whether these changes may have any effect on consumer acceptability.

## Figures and Tables

**Table 1:** Chemical composition of the alfalfa hay diet composition

Parameters	%
Dry matter	89.58
Ether extract	1.21
Crude protein	17.14
Ash	9.47
Crude fiber	18.18
Neutral detergent fibre	53.15
Acid detergent fibre	39.12
Acid detergent lignin	68.78

**Table 2:** Ingredients and chemical composition of the custom-formulated diet for control group (CG) and the experimental group (EL+)

Items	CG	EL+
Ingredients (%)		
Soybean meal	16.50	14.00
Fine bran	20.00	20.00
Barley meal	32.00	28.00
Corn meal	29.50	26.00
Extruded linseed	-	10.00
Vitamin and mineral premix	2.00	2.00
Chemicals composition of the concentrate (%)		
Dry matter	89.10	88.50
Ether extract	3.25	5.70
Crude protein	17.95	18.20
Ash	5.10	5.49
Neutral detergent fiber	12.55	13.32
Acid detergent fiber	5.95	5.44
Acid detergent lignin	1.16	1.39
Starch	48.59	43.88
Fatty acids (% total lipids)		
C14:0	0.60	0.20
C16:0	18.80	8.80
C16:1c9	0.30	0.10
C18:0	2.60	4.10
C18:1 c9	23.70	21.10
C18:1 c11	0.90	0.60
C18:2 c9,12	9.10	13.30
C18:3 c9,12,15	40.00	42.90
C20:0	0.40	7.00
Others	3.60	1.90

**Table 3:** Chemical composition of milk obtained from the control group (CG) and the experimental group (EL+)

Items	CG	EL+	p-value
Fat (%)	2.43±0.11	3.20±0.14	**
Protein (%)	3.19±0.13	3.50±0.17	*
Casein (%)	2.38±0.21	2.30±0.19	ns
Lactose (%)	3.81±0.18	4.30±0.25	*
Urea (mg/100 mL)	54.95±3.81	48.80±2.77	ns
SCC (×10^3^ cell/mL)	479±39	313±28	*
Milk production1) (mL)	1,209.00±38	1,300.00±51	*

The values are expressed as mean percentage±standard deviation.

SCC, somatic cell count.

1) Milk production for each animal for single milking.

* p<0.05;

** p<0.01;

ns, not significant.

**Table 4:** Chemical composition of cheese obtained from the control group (CG) and the experimental group (EL+) and analyzed after 60 (T_60_) days of ripening.

Items	CG	EL+	p-value
Fat (%)	18.48±1.77	15.91±0.19	ns
Protein (%)	21.20±0.85	21.55±1.77	ns
N (%)	3.31±0.12	3.38±0.28	ns
12% Trichloroacetic acid- soluble nitrogen (% N)	0.08±0.02	0.12±0.01	ns
Dry matter (%)	49.33±1.24	48.24±0.90	ns

The values are expressed as mean percentage±standard deviation.

ns, not significant.

**Table 5:** Acidic profile of bulk milk and ripened cheese T60 obtained from the control group (CG) and the experimental group (EL+)

Items	Bulk milk			Cheese (T_60_)		
	
	CG	EL+	p-value	CG	EL+	p-value
C4:0	2.61±0.48	3.01±0.19	*	1.49±0.70	4.09±0.53	**
C6:0	3.24±0.57	4.09±0.32	*	1.98±0.72	4.27±0.52	**
C8:0	4.11±0.16	4.72±0.36	*	2.65±0.82	4.62±0.45	*
C10:0	14.73±2.55	13.18±1.02	ns	10.67±2.75	14.60±0.98	ns
C12:0	9.19±1.03	8.63±0.66	ns	6.30±1.06	6.58±0.09	ns
C14:0	15.75±1.29	15.04±1.03	ns	15.74±0.67	14.29±0.98	ns
C15:0	1.08±0.09	1.01±0.08	ns	1.30±0.03	1.30±0.10	ns
C16:0	27.27±2.38	27.00±1.71	ns	28.64±2.56	20.22±0.46	*
C17:0	0.62±0.04	0.46±0.03	ns	0.72±0.52	0.70±0.30	ns
C18:0	3.42±0.12	3.80±0.19	*	8.54±1.21	8.36±0.64	ns
**SFA**	**82.02±0.93**	**80.94±0.56**	ns	**78.03±1.10**	**79.03±0.50**	ns
C14:1 c9	0.22±0.02	0.15±0.02	ns	0.60±0.01	0.57±0.24	ns
C16:1 c9	0.65±0.05	0.42±0.04	ns	0.34±0.08	0.70±0.61	ns
C18:1 t11	0.50±0.04	0.67±0.05	*	0.47±0.03	0.52±0.04	ns
C18:1 c9	13.30±0.14	13.60±0.12	*	17.45±2.71	16.39±0.52	ns
C18:1 c11	0.25±0.02	0.31±0.03	*	0.20±0.02	0.28±0.10	ns
**MUFA**	**14.92±0.95**	**15.15±1.05**	ns	**19.06±0.57**	**18.46±0.30**	ns
C18:2 c9,12	1.63±0.51	1.09±0.08	*	1.85±0.40	1.51±0.24	ns
C18:2 c9, t11	0.38±0.03	0.50±0.04	*	0.40±0.03	0.49±0.05	ns
C18:3 c9,12,15	1.05±0.54	2.32±0.16	*	0.66±0.74	0.51±0.28	ns
**PUFA**	**3.06±0.21**	**3.91±0.32**	*	**2.91±0.39**	**2.51±0.19**	ns
SCFA1)	5.85±0.53	7.10±0.26	**	3.47±0.71	8.36±0.52	**
MCFA2)	45.08±1.96	42.73±1.53	ns	37.26±0.89	41.96±0.47	*
LCFA3)	49.07±1.42	50.17±1.27	ns	59.27±1.91	49.68±1.29	**

The values are expressed as mean percentage±standard deviation. Fatty acids are expressed as percent of total fatty acids.

SFA, saturated fatty acid; MUFA, monounsaturated fatty acid; PUFA, polyunsaturated fatty acid; SCFA, short chain FA; MCFA, medium-chain FA; LCFA, long-chain FA.

1) C4:0 and C6:0.

2) C8:0 to C15:0.

3) C16:0 to C18:3.

* p<0.05;

** p<0.01;

ns, not significant.

**Table 6:** Aromatic profile of ripened cheese T60 obtained from the control group (CG) and the experimental group (EL+)

Items	VOCs	CG	EL+	p-value
Carboxylic acid	Formic acid	nd	5.56±0.51	-
	Butanoic acid	3.42±0.29	6.42±0.57	**
	Butanoic acid, 3-methyl	0.57±0.06	1.15±0.13	*
	Hexanoic acid	2.35±0.21	1.88±0.20	ns
	Heptanoic acid	0.34±0.04	nd	-
	Nonanoic acid	2.97±0.28	nd	-
	Decanoic acid	7.30±0.69	8.23±0.75	ns
	Dodecanoic acid	22.56±1.99	6.30±0.57	**
	Tetradecanoic acid	2.22±0.18	1.39±0.14	*
Aldehydes	Benzaldehyde	nd	2.32±0.21	-
	Nonanal	0.74±0.07	1.76±0.15	**
	Butanal, 3-methyl	nd	0.83±0.09	-
Alcohols	2.3-butanediol	4.11±0.37	3.30±0.28	*
	1-butanol, 3-methyl	7.17±0.62	22.62±1.96	**
	1-octanol	1.60±0.15	1.16±0.12	ns
	Phenylethyl alcohol	4.92±0.41	14.36±1.31	**
	1-decanol	0.79±0.07	1.08±0.11	ns
Esters	Ethyl hexanoate	6.33±0.59	6.43±0.58	ns
	Butanoic acid, pentyl ester	nd	0.55±0.06	-
	Butyric acid, 2-ethylhexyl ester	nd	0.34±0.04	-
	Propanoic acid, 2-methyl ethyl ester	2.66±0.22	1.79±0.14	ns
	Methyl octanoate	0.40±0.05	nd	
	Octanoic acid. Ethyl octanoate	6.01±0.56	5.13±0.47	ns
	Isopentyl hexanoate	nd	0.44±0.05	-
	Ethyl decanoate	6.39±0.58	nd	-
	Pentanoic acid 2.2.4-trimethyl-3-carboxyisopropyl isobutyl ester	nd	0.84±0.09	-
Lactones	δ-decalactone	0.48±0.06	0.53±0.06	ns
Ketones	Acetoin	1.03±0.01	nd	-
	2-heptanone	3.74±0.31	0.93±0.10	**
	3.5-octadien-2-one	nd	0.65±0.07	-
	2-nonanone	13.77±1.25	3.73±0.32	**
	2-decanone	0.53±0.06	0.29±0.04	*
	Benzil, methyl ketone	nd	0.41±0.05	-

The values are expressed as mean percentage±standard deviation; volatile compounds are expressed as percent of total volatile compounds.

VOCs, volatile compounds; nd, not detectable.

* p<0.05;

** p<0.01;

ns, not significant.
